# Leveraging Biochar Amendments to Enhance Food Security and Plant Resilience Under Climate Change

**DOI:** 10.3390/plants14131984

**Published:** 2025-06-28

**Authors:** Shakal Khan Korai, Punhoon Khan Korai, Muhammad Abuzar Jaffar, Muhammad Qasim, Muhammad Usama Younas, Muhammad Shabaan, Usman Zulfiqar, Xiaoshan Wang, Arkadiusz Artyszak

**Affiliations:** 1 Department of Grassland, College of Animal Science and Technology, Yangzhou University, Yangzhou 225009, China; khanshakal7@gmail.com; 2 Department of Soil Science, Faculty of Agriculture, Lasbela University of Agriculture, Water and Marine Sciences, Uthal 90150, Pakistan; punhoonkorai@gmail.com; 3 Department of Horticulture, Faculty of Agriculture, Lasbela University of Agriculture, Water and Marine Sciences, Uthal 90150, Pakistan; abuzar_jaffar@yahoo.com; 4 Microelement Research Center, College of Resources and Environment, Huazhong Agricultural University, Wuhan 30070, China; qrajput64@gmail.com; 5 Key Laboratory of Plant Functional Genomics of the Ministry of Education, Jiangsu Key Laboratory of Crop Genomics and Molecular Breeding, Agricultural College of Yangzhou University, Yangzhou 225009, China; usamaghias7@gmail.com; 6 Land Resources Research Institute, National Agricultural Research Center, Islamabad 45500, Pakistan; mshabaan@parc.gov.pk; 7 Department of Agronomy, Faculty of Agriculture and Environment, The Islamia University of Bahawalpur, Bahawalpur 63100, Pakistan; usman.zulfiqar@iub.edu.pk; 8 Institute of Agriculture, Warsaw University of Life Sciences-SGGW, Nowoursynowska 159, 02-776 Warsaw, Poland

**Keywords:** biochar amendment, climate change, crop production, resilient approach, carbon sequestration, GHG emissions, soil remediation, mechanisms

## Abstract

Climate change poses significant risks to food security and contributes to widespread soil degradation. Effective strategies are urgently needed to mitigate its impacts and ensure stable crop production and food quality. Biochar has shown strong potential to reduce greenhouse gas emissions, enhance carbon sequestration, and immobilize soil contaminants such as heavy metals and organic pollutants. These benefits can lead to increased crop yields, improved nutritional quality, and reduced uptake of harmful substances by plants. This review summarizes the possible mechanisms through which biochar influences the biochar–soil–plant interface, aiming to provide a comprehensive understanding of its multifaceted roles. Although positive effects of biochar on crop production are frequently reported, neutral or even negative outcomes have also been observed. Such adverse effects may be attributed to the presence of volatile organic compounds, free radicals, or heavy metals in certain biochars that inhibit plant growth. Additionally, biochar application has been found to reduce plant infections caused by pathogens, likely due to the presence of organic compounds that act as microbial inhibitors. A deeper understanding of the mechanisms by which biochar affects plant growth is essential for its effective use as a tool to combat climate change and enhance food security.

## 1. Introduction

Climate change, largely driven by human activities—primarily fossil fuel combustion and land use change—has increased the frequency of extreme temperature, precipitation, and flood events, alongside a persistent trend of global warming [1]. Anthropogenic greenhouse gas (GHG) emissions have risen from 27 (±3.2) to 49 (±4.5) gigatonnes of CO_2_-equivalents (CO_2_-eq) per year between 1970 to 2010, with fossil fuel combustion accounting for approximately 78% of this increase. In comparison, deforestation and land use changes contributed 12 Gt CO_2_-eq per year, representing around 22% of the anthropogenic emissions in 2010 [2]. Climate change is also exacerbating soil degradation, which is characterized by low organic carbon content, poor soil structure and water retention, elevated concentrations of heavy metals, and nutrient deficiencies, all of which pose serious threats to food security [3]. Furthermore, rising temperatures and shifting precipitation patterns are increasing crop susceptibility to pathogens, altering their distribution and abundance within changing habitats, thereby compromising crop health and food production [4,5,6,7]. All dimensions of food security—including food access, utilization, and price stability—are vulnerable to the impacts of climate change, as agriculture remains highly sensitive to climate variations [8]. Projections suggest that the global crop yield may decline by more than 5% by the 2050s if current trends continue [9,10].

Strategies are urgently needed to maintain a sustainable crop yield and to enhance crop nutritional levels under the scenario of climate change, one being to improve plant resistance to environmental stressors (adjustment from the internal plant body) and another to offset climate change (modification of external environmental factors). The former strategy can be realized by selecting cultivars and breeding productive crop varieties that are tolerant to the future warmer, drier, and higher [CO_2_] climate [11,12] and with modifying specific genes of crop varieties or species (Genetically Modified Organisms, GMOs) that can improve plant resistance to heat, drought, pests, and pathogens and to fix more CO_2_ [13]. Nevertheless, rather than enhancing plant resistance to environmental stressors from internal plant bodies by genetic modification, carbon-rich materials that are obtained from the slow pyrolysis of biomass residues under relatively low temperatures, named biochars, are widely known as a soil amendment technique used to potentially improve the external living conditions of plants and to offset climate change, together with many other co-benefits [14]. In comparison with the original feedstocks, biochars have high porosity, high surface areas, rich functional groups, and unique elemental compositions, characteristics which, as functions of the pyrolysis temperature, endow biochars with pivotal smart roles in terms of their interaction with soil components (such as natural organic matter, minerals, contaminants, and soil microbes) [15,16,17,18]. After atmospheric CO_2_ is captured through photosynthesis, living biomass is converted into agricultural and agroforestry residues. These residues can then undergo pyrolysis to produce both biochar and biofuel. Biochar contributes to long-term carbon sequestration in soil and supports increased primary productivity, thereby maintaining a sustainable supply of feedstocks for continued biochar production. Meanwhile, biofuel offers a renewable alternative to fossil fuels. Together, these processes contribute to achieving net negative CO_2_ emissions [19]. The amendment of soil using biochars can potentially enhance soil carbon sequestration, inhibit GHG emissions, improve soil structure and nutritional retention, immobilize soil contaminants, and increase crop yields and nutritional levels, which is promising in terms of ensuring sustainable food security [20,21,22,23].

**Figure 1:**
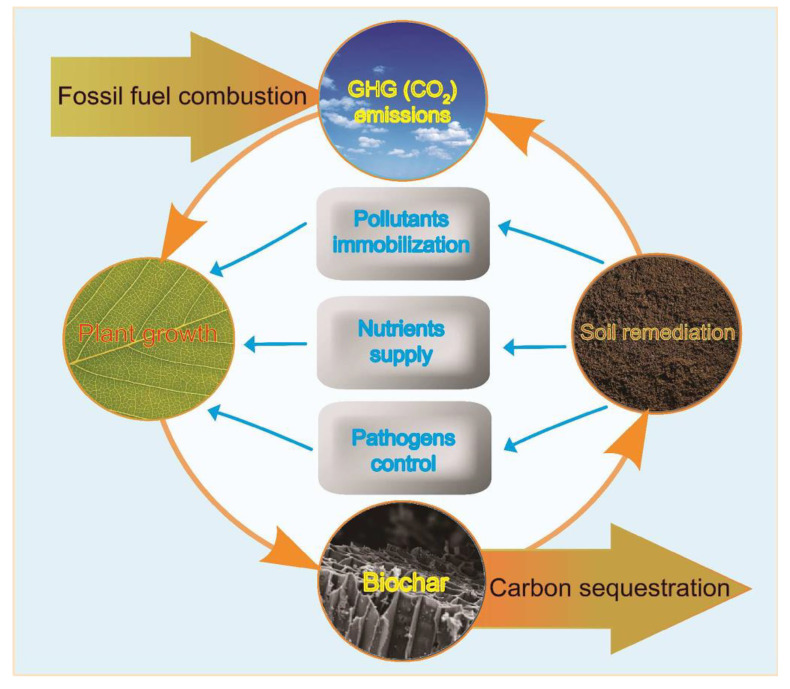
Schematic diagram illustrating the interactions of biochar with plant growth, soil remediation, carbon sequestration, and greenhouse gas (GHG) emissions, which form the basis of this review.

However, the effects of biochar amendments on plant growth vary widely, from positive to neutral and even negative, while the underlying mechanisms remain insufficiently understood. These effects are strongly influenced by biochar’s structural and chemical properties, which depend on the feedstock type and pyrolysis conditions, shaping the complex interactions at the biochar–soil–plant interface. Recently, novel applications of biochar, such as its potential in plant pathogen suppression, have emerged [24]. This review focuses on studies published between 2010 and 2024, a period marked by a rapid expansion in biochar research driven by its relevance to sustainable agriculture and climate change mitigation. Several recent reviews have addressed specific aspects of biochar, including modified biochar for soil improvement [25], biochar-based fertilizers [26], its role in suppressing plant pathogens [27], and applications in forestry systems [28]. While these studies offer valuable insights, the current review provides a comprehensive and integrated synthesis that emphasizes the mechanistic pathways by which biochar influences greenhouse gas (GHG) emissions, carbon sequestration, contaminant behavior, and plant health. Additionally, it introduces the novel concept of the GHG–plant–biochar (GBP) cycle (Figure 1), offering a unified framework to understand biochar’s multifunctional role. From the perspective of biochar–soil–plant interfaces, this review aims to explore how biochar contributes to climate change mitigation, enhances crop productivity and nutrient quality, regulates contaminant uptake, and supports pathogen control—ultimately highlighting its critical role in strengthening plant resilience under changing climatic conditions.

## 2. Biochars Mitigate Climate Change for Sustainable Crop Production

### 2.1. Effects of Climate Change on Crop Growth and Soil Degradation

**Figure 2:**
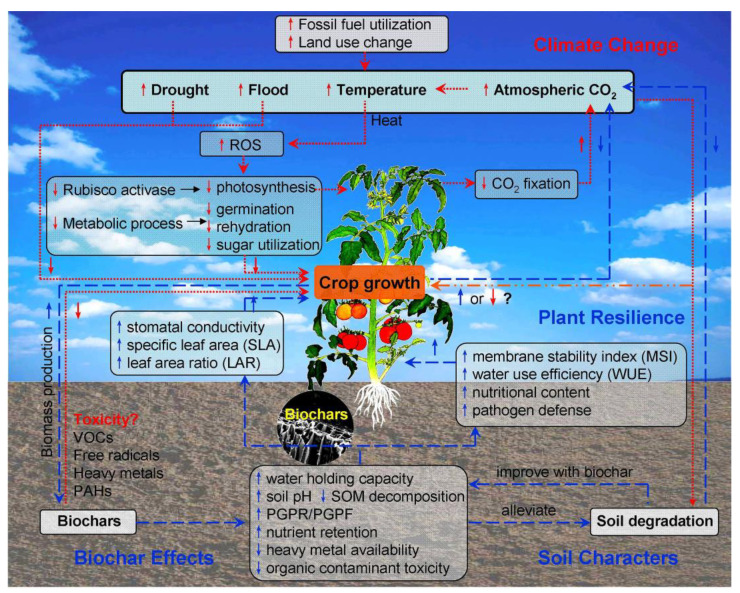
Schematic diagram showing the smart roles of biochar in offsetting climate change and enhancing crop resilience, with climate change primarily driven by fossil fuel use and land use change. Red dotted lines indicate these adverse effects. In contrast, blue dashed lines represent the positive contributions of biochar in offsetting climate change and promoting crop resilience. The orange dash-dotted line signifies the uncertain impact of elevated CO_2_ levels on plant growth, as outcomes may vary depending on interactions with other stressors such as heat and flooding. Red short arrows denote negative effects from specific processes, while blue short arrows indicate positive effects.

Climate change directly affects crop production by altering key environmental conditions such as temperature, precipitation, and atmospheric CO_2_ levels [29]. Under climate stressors, including heat, drought, flooding, and elevated CO_2_, the major constraints to improving crop productivity are poor soil quality, water scarcity, and low nutrient use efficiency [30]. Heat stress, in particular, can lead to the overproduction of reactive oxygen species (ROS), which damage the photosynthetic apparatus in plants (Figure 2) [31]. High temperatures inhibit the activity of Rubisco activase (Rca)—a crucial enzyme that restores the catalytic competence of Rubisco by removing tightly bound inhibitors from its active sites in an ATP-dependent process—thereby reducing photosynthetic efficiency and CO_2_ fixation [32]. In rice, heat stress can impair pollen germination by altering pollen morphology and disrupting key metabolic processes such as rehydration, resulting in reduced sugar activity and utilization [33]. The combined effects of extreme drought and heat can exacerbate plant stress, as stomatal closure to prevent water loss simultaneously limits the plant’s ability to dissipate heat [34]. On a global scale, extreme droughts and floods—often associated with El Niño events—can account for up to 35% of the variation in yields of wheat, oilseeds, and coarse grains [35,36].

Greenhouse gases, including CO_2_, methane (CH_4_), and nitrous oxide (N_2_O), have increased by about 90%, 47%, and 43%, respectively, from 1970 to 2010 [37], with an average global warming of 0.85 °C (0.65–1.06 °C) in the atmosphere from 1880 to 2012 [38]. Although elevated atmospheric CO_2_ concentrations alone have the potential to increase crop yields by 10–20% for C3 crops and 0–10% for C4 crops, interactions of elevated CO_2_ with changes in temperature and precipitation will cause complex impacts on crop yields that are not always beneficial, making the effects of climate change on agriculture complex and uncertain [39]. For example, increased water demands for growth with an elevated CO_2_ supply cannot be fulfilled under drier climate and can therefore limit crop growth; high-temperature attack on the flowering season may also diminish the benefits obtained from elevated CO_2_, making the net effects of elevated CO_2_ on crop yields not always positive. Additionally, the nutritional quality of food, including protein and micronutrients, can be negatively affected by the interaction of elevated CO_2_ with other aspects of climate change [40,41].

### 2.2. Biochars Mediate Greenhouse Gas Emissions, Carbon Sequestration, and Plant Responses

Biochars have the potential to mitigate GHG emissions, enhance carbon sequestration [42], and improve crop production [43]. The enhanced sequestration of atmospheric CO_2_ to the recalcitrant soil carbon pool with biochar application is partially through the reduction in SOC decomposition [44] and the inhibition of GHG emissions via alterations in soil biogeochemistry processes [45]. Annual net emissions of CO_2_, CH_4_, and N_2_O are estimated to be reduced by a maximum of 1.8 Pg CO_2_-C equilibrium (CO_2_-Ce) per year (that is, 130 Pg CO_2_-Ce per century) when sustainable biochars are produced and applied to soil (1 Pg = 1 Gt) [43].

While multiple biochar types have shown the potential to inhibit GHG emissions [46,47,48], a meta-analysis summarized the results from related publications, showing that biochars reduced soil N_2_O emissions by 54% in a laboratory and field study, with the key factors attributed to feedstock types, pyrolysis conditions, the C/N ratio, and the application rate of biochar [42]. Global CH_4_ emissions can be potentially mitigated with biochars as well, especially in flooded conditions and for acidic soils, issues which are more frequent under the background of global climate change [49]. Pyrolysis temperatures of biochar (rather than the feedstock types) can dominantly determine the influence of biochars on GHG emissions, since high-temperature biochar (700 °C) can lead to reduced GHG (CO_2_ and N_2_O) emissions, while moderate-temperature biochar (350 °C) has contrary effects, irrespective of feedstock type (including swine manure digestate biochar and willow wood biochar) [50]. Volatile matter content (VM), an easily mineralized C pool in biochar, can positively contribute to GHG emissions [51]. Improved soil porosity (i.e., aeration conditions) with biochar amendment can attribute to alleviated CH_4_ emissions, since the methanogenesis process is exclusively dependent on anaerobic conditions [49]. While a meta-analysis based on the published results of GHG emissions in response to biochar application indicates the main influencing factors, such as the biochar C/N ratio and soil moisture and pH [42,49], the underlying mechanisms should be further emphasized based on the interface interactions between biochar and soils. Possible mechanisms involved in the influence of biochars on soil GHG emissions should be clarified: (1) the liming effect of biochar that interferes with chemical and biological (microbial) processes, leading to alterations in GHG (N_2_O) emissions [42]; (2) the contribution of the volatile matter in the biochar (which represents the easily mineralized C pool) to GHG emissions, depending on the pyrolysis temperatures [51]; (3) the adsorption of NH_4_+ and NO_3_- in biochars with various sorption kinetics limits the N availability and changes the nitrification and denitrification processes [42]; (4) soil aeration conditions improved with biochar application govern the generation and diffusion of GHGs [42,49]; and (5) the inhibition of the microbial activities involved in C and N cycles via potentially toxic compounds in biochars, such as polycyclic aromatic hydrocarbons (PAHs), polychlorinated dibenzo dioxins and furans (PCDDs/Fs), and other volatile organic compounds (VOCs) [52,53].

In addition to GHG emissions inhibition, biochars can increase the recalcitrant soil carbon pool [54], a process which is not merely through the transformation of labile carbon from crop residues (and other feedstocks) into stable (aromatic) carbon structures with pyrolysis but also via the preservation of soil organic carbon due to the interaction of biochar with soil aggregates [55]. An increase in the soil carbon pool has the potential to offset fossil fuel emissions and to enhance crop yields and food security. In degraded cropland soils, an increase in the soil carbon pool by 1 t may increase crop yields by 20 to 40 kg per hectare (kg ha^−1^) for wheat, 10 to 20 kg ha^−1^ for maize, and 0.5 to 1 kg ha^−1^ for cowpeas [56]. Biochar can modify plant physiological features to improve plant resilience to climate change (Figure 2), such as by increasing the stomatal density and stomatal pore aperture of tomato leaf, even in non-irrigation conditions [57]. This can be attributed to the increased available water content of soils with biochar application by increasing the water content at field capacity and decreasing it at the permanent wilting point, which enables plants to extract more water from the soil prior to their wilting. As a consequence of the improved water status and stomatal conductance (gs) with biochar application, which is indicated by an improved relative water content (RWC), membrane stability index (MSI), and water use efficiency (WUE), tomato yield was increased by 6% under non-irrigated conditions [58]. Biochar application can also increase leaf allocation (which is associated with increased biomass), specific leaf area (SLA), and leaf area ratio (LAR, which indicates the leaf area relative to plant biomass) [59,60]. The benefits of biochar application on plant growth can be obtained via the preservation of soil pore water, thus maintaining a constant water moisture level and water holding capacity, and with the prevention of nutrients that are essential for plant growth from leaching during flood events [61].

However, the effects of biochar amendment on agricultural soil and crop yields have high heterogeneity, from positive and neutral to negative (Table 1), depending on the biochar type, application rate, soil characters, and plant species [62]. In some cases, a high application rate of some types of biochar may inhibit plant growth, possibly ascribed to micronutrient deficiencies induced by a high soil pH and high carbonate content caused by the biochar or due to reactive compounds being leached from the biochar [63]. Therefore, it is crucial to understand the functions of biochar in terms of promoting (or inhibiting) plant growth, including (1) the enhancement of plant nutrition, which guarantees a better development of plant biomass [64], (2) the stimulation of beneficial plant-growth-promoting rhizobacteria (PGPR) or fungi (PGPF) with biochar application, and (3) the hormesis effect on plant growth caused by low doses of biochar chemicals, which are phototoxic at high concentrations [65,66,67].

Appropriate amendment strategies should be considered to maximize the benefits of biochar amendment on crop yields and quality. Weathered and degraded soils characterized by a low cation exchange capacity, low soil organic carbon, low pH, and low clay content can obtain the maximum benefits in terms of crop quality improvements from biochar application [81]. For soils with low fertility, the application of biochars with an abundant ash content (that has high nutrient availability, P for example) and high porosity (which can provide more pore space for water retention) can enhance sunflower growth to greater extent than other biochar types [82]. Meta-analyses that quantitatively integrate the size of the effects of biochar application on crop yield based on the feedstock type, pyrolysis conditions, soil properties, and crop species could help to improve the knowledge of the maximum benefits that can possibly be gained from biochar application [14,83,84].

### 2.3. Variability and Standardization Challenges in Biochar Production and Application

Despite biochar’s promise in climate-smart agriculture, its properties can vary dramatically due to differences in feedstock, pyrolysis conditions, and production methods. Meta-analyses confirm that the feedstock type, such as wood, crop residues, or manure, has the strongest influence on biochar’s chemical and physical characteristics, including its carbon content, pH, surface area, nutrient concentrations, and contaminant-binding capacity [85,86]. Similarly, the pyrolysis temperature and heating rates critically affect biochar’s stability, porosity, aromaticity, and nutrient retention [87]. This heterogeneity often leads to inconsistent agronomic outcomes, with some biochars promoting yields and soil health and others performing poorly under similar field conditions. To address this challenge, certification systems such as the International Biochar Initiative (IBI) and European Biochar Certificate (EBC) have introduced detailed production and testing guidelines covering feedstock sourcing, pyrolysis emissions, and product characteristics to improve reproducibility and quality assurance. To fully realize biochar’s benefits, future research and practical deployment should embrace these standards and pursue optimized biochar formulations tailored to specific crop–soil–climate contexts.

## 3. Biochars Enhance Soil Fertility and Plant Nutrients

Although elevated CO_2_ levels can potentially enhance crop yields, the nutritional quality of crops may not increase proportionally due to soil degradation, which is further exacerbated by climate change. This can result in a dilution effect on the crop nutrient content, meaning that people actually receive less nutrients from the same amount of food, and larger quantities must be consumed to obtain the same nutrient levels obtained previously. With biochar application, improvements in soil nutrient status—particularly in terms of potassium (K), sodium (Na), calcium (Ca), magnesium (Mg), phosphorus (P), and nitrogen (N)—are frequently observed, and these improvements are essential for maintaining high nutritional quality in plants in addition to enhancing growth and biomass. Biochar application can be even more effective than the exclusive use of nitrogen fertilizers in improving total soil N, total S, and extractable nutrients such as P, K, Ca, and Mg [88]. In some cases, although no significant change in banana yield was observed with biochar amendment, nutrient uptake (e.g., K) increased, representing an improvement in food quality by supplying more nutrients per unit weight. The unique elemental composition and structure of biochars determine their functional properties, and understanding the structure–function relationships is key to recognizing the smart roles of biochar in improving soil quality and plant productivity (Table 2). The variable effects of biochar on plant nutritional contents are largely due to the ash content, which depends on the feedstock type and pyrolysis temperature. For example, peanut hull biochar, with a higher ash content than pine chip biochar, has shown more pronounced effects on improving soil pH and nutrient concentrations, thereby enhancing corn grain yields and stover biomass. A high ash content resulting from high-temperature pyrolysis increases the concentration of soil base cations and soil pH, while different pyrolysis temperatures can yield biochars with varying cation exchange capacities, even from the same feedstock type [89].

Nitrogen from plant-origin biochars may be less available than that from animal manure biochars; more specifically, wood biochars, which normally have a higher C/N ratio, are expected to increase N immobilization, while crop residue biochar, which has a relatively lower C/N ratio, increases N mineralization. The adsorption of NH_4_+ on the biochar surface and the increase in soil C/N ratio with the additional C input from biochar may lead to reduced N availability to plants, which may further result in a decreased leaf N concentration and chlorophyll content index (CII) [111].

However, the positive effects of biochar application may overcome the negative ones, such as N deficiency in plants (due to decreased N uptake and unaffected C content, resulting in an elevated C/N ratio in tomato leaf), and can increase plant nutritional levels, such as the titratable citric acid content and stable vitamin C content in tomato fruit. The re-release of nutrients adsorbed on biochars is a process dependent on time and environmental factors that enables biochar to act as a material that can release nutrients in a sustained manner. It may also take long time (years) for biochars to form aggregates with soil organic matter, a process which is beneficial for carbon sequestration [111]. While the benefits of biochars on crop production are widely reported, a large amount of biochar application (10%, for example) can also cause reductions in plant yields related to nutrient imbalances such as N deficiencies due to increased C/N ratios or K excess because of the extra supply of ash-derived K. Biochar with a high C/N ratio can be used for the main purpose of sequestering carbon due to low N bioavailability, while that with a relatively lower C/N ratio is more beneficial for maintaining a high crop yield without causing obvious N limitation [112].

## 4. Biochars Restrain Soil Contaminants to Guarantee Food Safety

### 4.1. Climate Change Increases Soil Contaminant Availability and Plant Uptake

Heavy metals and organic contaminants can be strongly bound to soil organic matters for centuries, and they can be mobilized into a bio-available form with increased temperature, soil acidification, drainage, and soil erosion, becoming a risk for food safety [14,60]. The fixation and deposition of the increased N_2_O emissions from fossil fuel combustion cause soil acidification, lead to the release of toxic Al affecting fine root growth and base cation (such as Ca^2+^, K^+^, and Mg^2+^) uptake by plants, and may increase the mobility of heavy metals [113]. Environmental temperature and precipitation patterns affect the diffusion of weakly sorbed contaminant compounds and the sorption of moderately and strongly bound compounds. Warmer soil enhances the decomposition of soil organic matter (SOM), resulting in the release of pollutants (heavy metals and organic contaminants) that were formerly bound therein [56,114]. A temperature increase by 10 °C can reduce the half-life of pesticides in soils by 60% and enhance their volatilization, which can potentially expose new plant populations to their toxic effects [115]. It is not only the mobility and bioavailability of heavy metals that can be accelerated with higher temperatures due to the decomposition of SOM but the plant physiological process through which heavy metals are taken up and accumulated by plants can also be enhanced. Nevertheless, increased temperatures will also accelerate contaminant degradation. The balance between the release of contaminants from SOM and their degradation, leaching, and uptake by plants should be estimated to evaluate their potential risks under a changing climate [116].

Plants growing in degraded soils are more susceptible to climate change, especially in soils with high available metal (loid) concentrations, low pH, and high salinity. A decrease in soil moisture content from 50% to 30% in terms of the soil water holding capacity enhanced the susceptibility of organisms to heavy metals in polluted soils but not in a healthy forest soil. By increasing the organic acid and DOC contents in the soil solution, reducing pH in the rhizosphere, and increasing the Cd/Zn-DOM (dissolved organic matter) complex fraction in the soil solution (by around 8%), elevated CO_2_ can enhance the mobility and bioavailability of Cd and Zn, thereby increasing their uptake by some plant species (e.g., a hyperaccumulator, Sedum alfredii) [117]. Furthermore, elevated CO_2_ may alleviate oxidative damage in plants caused by heavy metals, resulting in an enhanced accumulation of metals such as copper (Cu), iron (Fe), manganese (Mn), lead (Pb), and zinc (Zn) in plant tissues without significantly inhibiting plant growth [118]. While this response can be advantageous for phytoextraction and phytoremediation, it also poses potential risks to food safety if the edible parts of crops accumulate elevated levels of heavy metals.

### 4.2. Biochars Restrain Crop Uptake and Toxicity of Soil Contaminants

For soils contaminated with organic contaminants (such as PAHs) and heavy metals, biochar application can alleviate the toxicity of soils to plants, which is attributed to the immobilization of the bioavailable fractions of organic contaminants and heavy metals; however, the effects are dependent on the soil properties, biochar type, and application rate [119]. A stimulating effect on the root growth of *Lepidium sativum* was observed with willow biochar application to a soil from a bitumen plant area (contaminated with PAHs) at rates ranging from 0.5% to 5%, while at some application rates with wheat biochar, a positive effect was not found [120]. It is critical to understand the multiple mechanisms involved in the interactions between biochar and contaminants in order to better tailor biochar production for specific purposes and to more accurately predict the effects of a given application.

#### 4.2.1. Biochar’s Effects on Bioavailability and Plant Uptake of Heavy Metals

**Figure 3:**
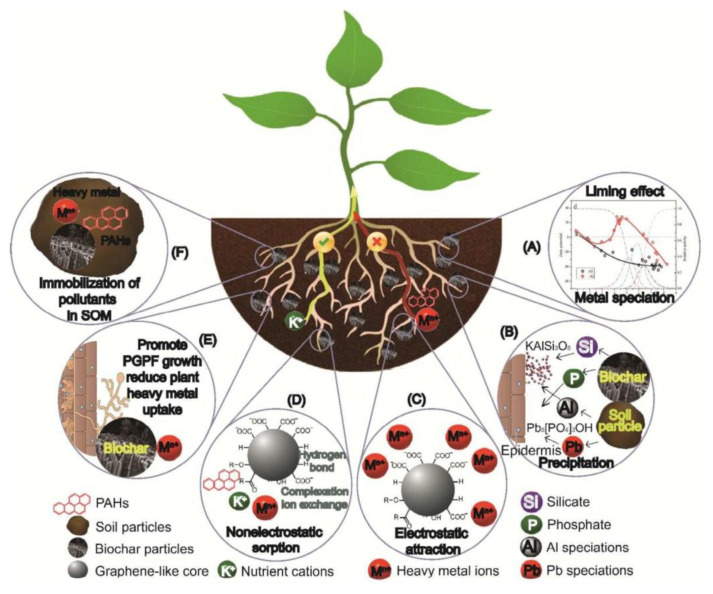
Schematic diagram showing the mechanisms by which biochar can potentially immobilize heavy metals and organic pollutants (PAHs as a representative) and reduce plant uptake. (**A**) Biochar induces a liming effect, altering heavy metal speciation and reducing toxicity. (**B**) Silicon and phosphorus minerals released from biochar can form complexes with heavy metals, leading to their accumulation on the root epidermis and preventing translocation from roots to shoots. (**C**) Electrostatic attraction between biochar functional groups on its graphene-like core and metal cations aids in heavy metal immobilization. (**D**) Non-electrostatic interactions—such as hydrogen bonding, ion exchange, and complexation—further reduce metal bioavailability. (**E**) Biochar-mediated immobilization of heavy metals supports the growth of plant growth-promoting rhizobacteria (PGPR) and fungi (PGPF), which benefit plant health. (**F**) Biochar increases soil organic carbon (SOC), enhancing the sorption of heavy metals and organic pollutants in soil organic matter (SOM).

The possible mechanisms involved in biochar’s effects on heavy metal immobilization and plant uptake include the following (Figure 3): (1) Increased soil pH with biochar amendment, in terms of limiting effects, can regulate heavy metal speciation, decrease their mobility, and reduce their uptake by plants. (2) The precipitation of heavy metals with crystal formation on biochar due to the interaction between the minerals in the biochar and the heavy metals can effectively reduce heavy metal solubility and plant uptake [121]. (3) Biochar can enhance the negative charge of soil, providing an electrostatic force between heavy metal ions and surface functional groups such as carboxyl and phenol in biochar [122]. (4) Biochar can enhance non-electrostatic forces, such as ion exchange between the biochar surface functional groups (such as carboxyl groups) an heavy metal ions [111] and the complexation of heavy metals with the carboxyl (-COOH) or phenolic -OH groups of biochar [48,123]. (5) Enhanced soil nutrient retention with biochar amendment can increase the plant uptake of nutrients (Ca^2+^), which can compete with heavy metals (Cu^2+^) for adsorption sites and, in turn, reduce the plant uptake of heavy metals (for example, via ion exchange, as indicated in Figure 3D) [124]. (6) An increased SOC content with biochar application can provide effective adsorption sites for heavy metals, reducing the heavy metal content in pore water, and as a result, it can restrain the plant uptake of hazardous metals [125]. (7) With the restraint of heavy metal bioavailability, biochar can increase the proportion of plant-growth-promoting bacteria (or fungi), which, when synergistically combined with biochar, can enhance the edible tissue growth of vegetables (e.g., Chinese cabbages and radishes) and reduce the Cd and Pb uptake in their edible tissues [126]. The sorption of heavy metals and nutrients by biochars is not selective; thus, the strong sorption capacity of biochar for heavy metals, which is beneficial for heavy metal immobilization, could possibly simultaneously be accompanied with nutrient deficiency in plants. Therefore, it is essential to apply biochars with specific features according to the target purposes, and the auxiliary application of fertilizer can be helpful in certain circumstances to ensure multiple functions of biochar. Key roles of phosphate and silicate from biochars in heavy metal immobilization have been found (Figure 3B) [127,128,129]. For example, biochar inhibited the phytoavailability of Pb in maize (*Zea mays* L.) with the formation of Pb–phosphate in the soil, reducing the Pb concentration in shoots [130]. As a soil remediation, biochar can alleviate Al toxicity in soils and in plants, primarily by reducing the amount of soil Al^3+^ with the precipitation of Al by silicate particles (in the form of KAlSi_3_O_8_) and via adsorption by oxygen-containing organic components [131], thus preventing the migration of Al to the plants. In addition, a novel mechanism has been found for the alleviation of Al toxicity in plants with the application of Si-rich biochar (rice straw biochar). The Si released from the biochar can form a complex with Al on the root epidermis, which effectively prevents the transportation of Al from roots to shoots, reducing the toxicity of Al to the aboveground biomass of wheat seedlings [94].

The adsorption of metal ions on biochar can be more profound in degraded soils with poor quality, such as soils with a low pH and SOC content, because soils with a low pH normally have a high mobility of heavy metal ions, which can be effectively adsorbed onto the carboxyl and phenol functional groups of biochar, and soils with a low native SOC content can limit competition for the sorption sites of biochar via soil organic matter with heavy metals [132,133]. Furthermore, with a reduction in the heavy metal concentration in plants, the heavy metal stress on the plants can be alleviated, resulting in increases in plant biomass and nutrient content (Table 3), which is beneficial for food safety [134]. Simultaneously, by reducing the uptake of heavy metals by plants, biochar can alleviate nutrient deficiencies caused by heavy metal stress. This is because the inhibition of proton-pumping ATPase activity on the plant plasma membrane—induced by heavy metals—can be mitigated through the adsorption and precipitation of heavy metals on biochar surfaces, thereby ensuring the uptake of essential plant nutrients [128].

#### 4.2.2. Biochar Effects on Organic Pollutant Toxicity to Plants

The fate of organic contaminants in soil with biochar application includes sorption, desorption, leaching, volatilization, and dissipation (degradation), and bonding organic contaminants with biochar can reduce their bioavailability (Figure 3) [150,154]. The mechanisms for the sorption of organic contaminants in biochar include the following: (1) the partition to the amorphous carbon component of biochar (moderate temperature), (2) physisorption via pore diffusion, π*-π electron donor–acceptor interaction, H-bonding, and van der Waals dispersion forces, and (3) chemisorption via chemical bonding with phenolic and amine groups [155,156]. Biochar application can reduce the bioavailability of organic pollutants, alleviate their toxicity to plants, and promote plant growth (Table 3). Sorption mechanisms are determined by the biochar’s properties as a function of the pyrolysis temperature and feedstock types [15]. The adsorption of antibiotics (sulfamethazine, SMT) by plant residue (*Sicyos angulatus* L.) biochar pyrolyzed at 300 °C and 700 °C occurs mainly through electrostatic interactions, with the possible participation of other mechanisms such as hydrophobic, hydrogen bonding, and π-π interactions [157,158]. Due to the different sorption mechanisms of PAHs with variable molecular sizes to biochars, high-molecular-weight PAHs can be more effectively reduced than low-molecular-weight PAHs with biochar sorption. Since PAHs with a higher molecular size are considered to be more carcinogenic, their high sorption efficiency with biochars is beneficial for food safety [146]. The adsorption of persistent organic pollutants (POPs) by biochar could be the main pathway for the dissipation of such organic contaminants, and their adsorption can limit plant uptake and microbial degradation within the rhizosphere [149,159].

With respect to herbicides, which are used in agricultural activities for weed control, their efficacy, their persistence in the environment, and the bioavailability of their residues to plants are crucial aspects in evaluating the efficiency of a sorbent application such as biochar. Biochar application can enhance the immobilization of atrazine and acetorchlor in soil [101,160]. Treatment with biochar could extend the half-life (from 5.2 days to 21.5 days) of an ionizable herbicide (4-chloro-2-methylphenoxy acetic acid, MCPA) in soil due to its sorption in the biochar [150]. An amorphous biochar (400 °C, from wood and grass) showed the highest sorption parameters of two herbicides (Norflurazon and Fluidone) compared to biochars produced at other pyrolysis temperatures (both lower and higher). As revealed by the sorption isotherms of these herbicides, high-temperature biochars (500 °C and 600 °C) with a decreased Koc of herbicides exhibited adsorption mainly due to aromatic carbon groups rather than the linear partition that dominated in low- and moderate-temperature biochars [161]. The high adsorption capacity of biochar can be attributed to its high SSA [105] . However, the consequently reduced bioavailability of herbicides may inhibit the efficacy of weed control, leading to a need for a higher application rate of herbicides, which is not profitable for sustainable agriculture. From this viewpoint, biochars with a low SSA are recommended for the purpose of maximizing the benefits in terms of both pesticide control and agricultural benefits [120,162].

#### 4.2.3. Potential Toxicity of Biochar on Plant Growth

In spite of the benefits of biochar application on pollutant immobilization, in some cases, however, increased toxicity of soils to the plants was also found after biochar application to the soil [163]. This raises a concern in terms of the toxicity of biochar per se on plant growth (Figure 2). It was found that the toxicity of biochar in term of PAHs and heavy metal (and metalloid) contents were dependent predominately on the heat treatment temperatures (HTTs) and feedstock types [164]. Short-chain carboxylic acids and phenolics detected in the leachates of biochar (forestry residue wood biochar), including the known phytotoxic acetic acid, butyric acid, 2,4-di-tert-butylphenol, and benzoic acid, can primarily explain the negative plant growth response (for example, biomass and leaf area) to biochar application, indicating the potential toxicity of biochar to plant growth. The pre-treatment of biochar is thus advocated for before application, such as water washing, which can alleviate the inhibition of plant growth due to the phytotoxic organic compounds in biochar [165]. In cases where biochar amendment can rather support metal uptake (Pb) by potentially accumulating plants (mustard, for example), biochar combined with this plant species can be used for phytoextraction treatment to remove soil heavy metals [166].

Nevertheless, in some cases, despite the high content of available potentially toxic elements (PTEs) in biochar pyrolyzed at 750 °C, no correlation was found between the available PTEs and the inhibitory effects on plant growth (e.g., cress seedlings) [167,168]. This suggests that PTEs may not be the primary inhibitors of growth in certain plant species and that the toxicity mechanisms of biochar can vary depending on its properties, the plant’s physiological characteristics, and soil types. Additionally, possible toxicity resulting from free radicals generated during the pyrolysis process presents another potential pathway for phytotoxic effects at the biochar–plant root interface. Identifying the mechanisms underlying biochar phytotoxicity remains a critically important area of research [169,170].

## 5. Biochars Enhance Plant Resistance Against Pathogens

### 5.1. Climate Change Induces Severer Pest and Pathogen Caused Plant Disease

Alterations in temperature, rainfall, and CO_2_ levels under climate change are critical factors that influence the distribution and growth patterns of pests and pathogens by increasing the number of generations and population growth rates, reducing generation times and overwintering mortality, and altering crop–pest synchrony [8,171]. Climate fluctuations also affect the contamination phase of mycotoxin production by fungi, such as aflatoxins. Dry and hot conditions favor contamination during crop development, while wet and warm conditions favor the infection phase after crop maturation. High temperatures and drought stress also increase the incidence of plant diseases caused by *Fusarium verticillioides*, a fungal species that produces the carcinogenic mycotoxin fumonisin and causes rot in various plant parts. These fungal pathogens can significantly reduce crop productivity, leading to an estimated pre-harvest loss of 8.6% in global maize production [172].

Climate change influences the quantity of mycotoxin-producing fungi [173] and can cause increased susceptibility of both C4 crops and C3 crops to pathogens, including *Fusarium verticillioides* (maize), *Magnaporthe oryzae* (rice), and *Fusarium pseudograminearum* (wheat) [174,175,176]. The presence of mycotoxins is impacted by increased temperature, such that a 2 °C increase scenario can predict a food safety issue arising from aflatoxin B-1 risk [177]. A warmer climate may increase the number of generations and population growth rate of soil-borne fungal pathogens by reducing generation time and overwintering mortality during mild winters, leading to more frequent root infections. Wet conditions accelerate the germination and spread of fungal spores, as well as the proliferation and colonization of fungi and bacteria [178]. Elevated CO_2_ (800 ppm, about a 2-fold increase compared to the current concentration) is largely responsible for the increased plant susceptibility to the mycotoxigenic fungi *Fusarium verticillioides* and attenuated maize defenses, causing aggravated maize stalk and kernel rot, leading to reduced maize production [176]. With respect to plant resistance, heat or drought stress can reduce phytoalexin production, resulting in host susceptibility of plants to pathogens, such as increased compromise for maize kernel integrity and hull cracking of pistachios [179].

### 5.2. Essential Role of Biochars on Plant Resistance Against Pathogens

Biochar is able to enhance plant resistance to pathogens by modifying the immune system of plants and altering microbial-to-plant communications in the rhizosphere [67,171,180]. This modification can be regulated by plant secretion of microbial active substances (e.g., a small intracellular protein, PR10, which can exhibit antimicrobial activity in vitro against bacteria, fungi, and viruses) and the adsorption of such substances by biochar, a process which can interfere with the communication between plant roots and plant-growth-promoting rhizobacteria (PGPR) or fungi (PGPF) [180]. The enhanced resistance of plants to microbial pathogens can also be induced by direct toxic effects of certain biochar volatile organic compounds (VOCs) on microbial pathogens [105]. A high application rate (3%) of biochar is able to induce a higher defense ability to disease caused by pathogens compared to a low application rate (1%) [180], possibly due to higher amounts of microbial inhibitors such as some VOCs extracted from biochar [101]. Both pepper and tomato plants receive benefits from biochar application in terms of resistance against two foliar fungal pathogens (Botrytis cinerea and Leveillula taurica) [171]. Additionally, this effect has been found with various biochar types, such as wood biochar and greenhouse waste biochar, which both stimulated a range of general defense pathways of strawberry plants and, as a result, suppressed anthracnose disease caused by the fungal pathogens Botrytis cinerea, Colletotrichum acutatum, and Podosphaera apahanis [180]. It should be noted that plant defense and disease reduction, as the positive effects of biochar amendment, are not always correlated with plant growth responses, which can be considered as trade-offs between defense and plant growth [181,182,183]. The direct toxicity seems not sufficient to solely explain the disease suppression mechanism with biochar application, seeing that there is a rule-of-thumb inverted U plot between the biochar dose and the inhibition of soil-borne pathogens [105]; for instance, the intermediate biochar doses (0.5% and 1%), compared to a high dose (3%), showed the most promising suppression of the damping-off of common bean caused by Rhizoctonia solani. It is possible that a high dose of biochar may release a larger amount of chemicals or free radicals that can possibly damage plant roots [170,184].

However, an enhanced defense of foliar fungal pathogens (Botrytis cinerea and Leveillula taurica) with citrus wood biochar at application levels from low to high (1%, 3%, and 5%) was consistently found as well [171,185]. We hypothesize that the variable results could have been caused by the different chemical compositions of the biochars as a function of the pyrolysis conditions and feedstock types, as well as the different pathogen and plant susceptibilities to these components. In addition to the effects of the biotoxins and the hormone-like compounds from biochar, influences on soil-borne pathogens can be related to nutrient supply, pH alterations, and the adsorption capacity of biochar, as well as changed soil physical characteristics that will have a profound effect on soil microbial communities and functioning, which may change the activity and abundance of soil pathogens. As an emerging research field, the potential roles of biochar on plant pathogen defense and suppression should be emphasized based on the investigation of various biochar types and characteristics on plant defenses against different pathogens [105].

## 6. Conclusions and Perspectives

Biochars serve multiple functions in mitigating greenhouse gas (GHG) emissions, enhancing carbon storage, improving plant nutritional content, increasing crop yields, and strengthening crop resilience to climate-change-induced variables, with most findings being positive (Table 1). Certain characteristics of biochar are crucial for its environmental effects, including pH, volatile matter (VM), specific surface area (SSA), carbon and nitrogen content (including the C/N ratio), ash content, elemental composition, carbon structure, and more. A thorough understanding of the underlying mechanisms in biochar–soil–plant interactions is critically important. This review summarizes the possible mechanisms by which biochar contributes to climate change mitigation and plant growth enhancement, with an emphasis on the multifunctional roles of biochar at the biochar–soil–plant interface. Biochar can potentially reduce GHG emissions and enhance carbon sequestration by inhibiting soil organic matter (SOM) decomposition in warmer soils through SOM sorption and alterations in soil biogeochemical processes. The GHG–plant–biochar (GPB) cycle proposed in this review can result in a net negative carbon loss by simultaneously enhancing carbon sequestration and reducing GHG emissions (Figure 1). Improved soil physicochemical conditions resulting from biochar application can enhance water and nutrient uptake by plants, ultimately benefiting food quality. Enhanced plant physiological traits—such as improved water use efficiency and membrane stability—serve as evidence of increased plant resilience to climate change due to biochar application. Nutrient supply from biochar varies with biochar type and is primarily influenced by the pyrolysis temperature, mainly due to differences in ash content. The effective sorption of heavy metals by biochar ensures a significant reduction in plant uptake (Table 3), which can further alleviate nutrient deficiencies caused by heavy metal stress. Growing evidence suggests that biochar application can enhance plant resistance to pathogen-induced diseases, highlighting a promising role for biochar as a pathogen inhibitor and opening new avenues for exploring its potential mechanisms of action.

However, future research must consider the trade-offs between the multiple functions of biochar in promoting plant growth and improving soil quality. The benefits of biochar in crop production, nutrient uptake, carbon sequestration, and GHG emission reduction may not all be realized simultaneously with a single application. Biochars can exert dual effects on plant growth. For example, while the sorption capacity of biochar may immobilize pollutants, it can also limit short-term nutrient availability to plants. Additionally, volatile organic compounds (VOCs) and free radicals in biochar may suppress soil borne pathogens and reduce plant infections, but under certain conditions, they may also harm root development. These dual functions raise critical questions: (1) Under what conditions do the benefits of biochar amendment outweigh the potential phytotoxic effects on plant growth? (2) How can the drawbacks—such as nutrient deficiencies—be minimized while maximizing the benefits, including carbon sequestration, pollutant immobilization, and improved crop production? To address these questions, a comprehensive understanding of the mechanisms underlying the systemic effects of biochar on plant growth is essential. In particular, elucidating the composition–structure–function relationships of biochars represents a key research direction for uncovering these mechanisms.

## Figures and Tables

**Table 1:** Effects of soil biochar amendment on crop yields and nutritional content, along with associated changes in soil characteristics.

Biochar Feedstock’s	Crops	Soil Types and Variations	Effect on Crop Yield	Reference
**Crop residue biochar**				
Rice husk and cotton shell mixture biochar (400 °C).	Tomato	Sandy loamy soil with different irrigation treatments.	Fresh fruit weight ↑ Improved by 20%, 13%, and 6% under high to low water conditions, titratable citric acid concentration. ↑ Enhanced.	[68,69]
Rice paddy husk biochar (10 t ha^−1^).	Banana	↑ Enhanced soil potassium, magnesium, sodium, and phosphorus. ↑ Enhanced % carbon and % nitrogen.	Crop yield not measured.	[70,71]
Peanut hull biochar (400 °C).	Corn	Loamy sandy soil. ↑ Improved soil nitrogen, phosphorus, potassium, sulfur, calcium, magnesium, and soil pH.	Corn grain yield, stover biomass, and corn tissue potassium concentration ↑ Increased.	[72,73]
Green waste biochar (500 °C).	Wheat	Haplic calcisol (calcareous soil). No significant influence on soil organic carbon, dissolved organic carbon, microbial biomass carbon, and microbial biomass nitrogen.	Slightly ↓ Reduced wheat grain yield, biochar effect inferior to di-ammonium phosphate fertilizer.	[74,75]
**Wood biochar**				
Wood biochar.	Banana	↑ Enhanced soil pH.	↑ Improved crop nutrient (potassium) uptake, no change in fruit production.	[76,77]
Willow wood biochar (500 °C).	Banana and papaya	↑ Increased soil pH, potassium, calcium, cation exchange capacity, NH_4_^+^-N, NO_3_^−^-N, % carbon of red clay soil.	Average fruit diameter ↑ Enhanced.	[78]
Willow wood biochar (500 °C).	Banana and papaya	Water content, pH, sodium, % carbon, carbon/nitrogen, and carbon stock of red chromosol.	Slightly ↑ Increased average number of fruit per tree.	[78]
Pine chip biochar (400 °C).	Corn	Loamy sandy soil, ↓ Reduced soil calcium, and pH.	Corn grain yield and tissue potassium, sulfur, and magnesium concentration ↑ Increased.	[72,73]
Hard wood biochar (slow-pyrolysis charcoal at (500 °C) and gasification coke at (1100 °C), soft wood biochar (flash-pyrolysis biochar at (450–550 °C) (15 g kg^−1^ ≈ 45 t ha^−1^).	Corn (*Zea mays* L.)	Sandy Ap horizon of a haplic fluvisol with poor cation exchange capacity and moderate water holding capacity, ailty Ap horizon of a gleyic luvisol with medium potential cation exchange capacity and very high water holding capacity. ↑ Improved soil total carbon and black carbon fraction determined with benzene polycarboxylic acids. Gasification coke ↑ Increased sandy soil pH. Flash-pyrolysis char ↓ Reduced silty soil pH. No influence on soil water holding capacity or aggregates.	Slightly but not significantly ↓ Reduced biomass and corn yield, no influence on nutrient contents or functional traits such as leaf area ratios and specific leaf area. Flash-pyrolysis char ↓ Suppressed the germination of maize kernels.	[79]
Hard wood biochar (slow-pyrolysis charcoal at (500 °C) (100 g kg^−1^ ≈ 300 t ha^−1^).	Corn (*Zea mays* L.)	Silty Ap horizon of a gleyic luvisol with medium potential cation exchange capacity and very high water holding capacity. ↑ Enhanced soil water holding capacity. ↑ Improved soil carbon/nitrogen (to a high value of 80) and plant available potassium.	↓ Reduced corn yield and calcium content. ↑ Increased magnesium and carbon/nitrogen ratio in leaf biomass.	[79]
Citrus wood biochar.	Pepper and tomato	Fertigated soil-less media	↑ Enhanced leaf area, canopy dry weight, number of nodes, and yield of buds, flowers, and fruit of pepper plant. ↑ Increased plant height and leaf size, no effects on flower and fruit yield.	[80]
**Other biochar types**				
Water-washed gasification coke produced at 1100 °C (15 g kg^−1^ ≈ 45 t ha^−1^).	Corn (*Zea mays* L.)	Silty Ap horizon of a gleyic luvisol with medium potential cation exchange capacity and very high water holding capacity. ↑ Increased soil phosphorus and magnesium, ↓ Reduced potassium.		[79]

**Table 2:** Biochar elemental composition, structure, and related properties contribute to increased plant nutrient availability, enhanced crop growth, immobilization of toxic pollutants, reduction in GHG emissions, and reinforcement of carbon sequestration.

Composition	Related Properties	Potential Functions	References
**Elemental composition**			
C	Carbon stability, sorption capacity	Aromatic carbon increases carbon sequestration and adsorption of pollutants. Amorphous carbon can be utilized by microbes as a carbon source and is responsible for the partition of pollutants.	[90,91]
Si	Carbon stability, nutrient concentration	Maintains a stable carbon structure with the formation of a C-Si complex during pyrolysis. Alleviates plant Al uptake with the formation of a Si-Al complex on the plant root cell epidermis. Supplies Si for plants with the release of soluble Si from biochar.	[92,93,94]
N	Carbon stability, nutrient concentration, carbon source quality	As a soil fertilizer to release nutrients adsorbed on the biochar surface during aging in a sustained manner for plant nutrient uptake. The C/N ratio of the biochar determines C and N bioavailability to soil microbes and plants.	[95]
P	Nutrient concentration	As soil fertilizer to release nutrients adsorbed on the biochar surface during aging in a sustained manner for plant nutrient uptake.	[95]
K	Nutrient concentration	As soil fertilizer to increase plant K uptake.	[77]
Atomic H/C	Nonpolarity, sorption capacity, carbon stability	Causes a decrease in atomic H/C indicated in the aromatic structure and the recalcitrant nature of biochar. Causes an increase in the adsorption capacity of biochar.	[96]
Atomic O/C	Nonpolarity, sorption stability	Causes a decrease in atomic O/C indicated in the aromatic structure and the nonpolarity of biochar.	[91,97]
C/N	Carbon stability, carbon source quality	Causes a high C/N decrease and microbial decomposition because of a lack of N supply, thus enhancing carbon stability. High C/N represents poor nutrient quality for plant uptake.	[98]
Ash content	pH, nutrient concentration	Has a liming effect to increase the soil pH. Supplies nutrients for plant growth.	[73,99]
**Structure**			
Aromatic carbon	Carbon stability, sorption capacity	Increases carbon sequestration, decreases soil microbial mineralization. Causes the sorption of organic compounds (including pollutants). Decreases GHG emissions.	[90,100,101]
Surface functional group	Sorption, pH	Causes the sorption of nutrients and heavy metals and some organic compounds. Reduced acidic functional groups (such as -COOH) with pyrolysis through increased temperature can increase biochar pH.	[102]
Porosity	Density, pore volume	High porosity improves soil aeration conditions, which modifies the GHG emission process. Enhances soil water retention, which ensures plant resistance to dry weather. Provides microbial inhibition.	[103,104]
SSA	Sorption, CEC	Adsorbs nutrients (as soil fertilizer) and organ pollutants. Forms a soil aggregate to protect SOC from decomposition.	[105,106]
Negative surface charge	Sorption, CEC	CEC dependent on environmental pH controls the long-term release of nutrients from biochar or sorbed on biochar from soil.	[107,108,109]
VM and VOCs	Carbon stability, potential toxicity	VM represents the labile carbon in biochar. VOCs may inhibit soil-borne pathogens and improve plant growth.	[80,110]

SSA = specific surface area, CEC = cation exchange capacity, SOC = soil organic carbon, VM = volatile matter, VOCs = volatile organic compounds.

**Table 3:** Effects of biochar amendment on contaminant availability (heavy metals, hazardous metalloids, and organic pollutants) and plant uptake, along with potential mechanisms, as reported in the cited references.

Biochars	Pollutants	Plants	Soil Type	Bioavailability	Plant Uptake	Possible Mechanisms	References
**Heavy metals**							
Oak wood biochar 400 °C	Pb	Maize		**↓** Decreased Pb bioavailability	**↓** Decreased Pb accumulation in maize shoots	Formation of Pb–phosphate in soil, immobilizing Pb	[135]
Wheat straw biochar 350–550 °C	Pb	Rice	paddy soil	**↓** Decreased extractable Pb	**↓** Decreased Pb only in roots	Precipitation and adsorption, bound to mineral phase of Al, Fe, and P	[136]
Wheat straw biochar 350–550 °C	Cd	Rice	paddy soil	**↓** Decreased extractable Cd	**↓** Decreased Cd in rice grain, shoots, and roots, **↑** Increased rice yield	Precipitation and adsorption, bound to mineral phase of Al, Fe, and P	[136]
Willow wood biochar 500 °C	Cd, Pb, and Zn	Spinach	modal chernozem	**↓** Decreased Cd and Zn mobility	**↓** Decreased Cd, Pb, and Zn uptake, **↑** Increased biomass	Heavy metal adsorption by biochar	[137]
Willow wood biochar 500 °C	Cd, Pb, and Zn	Mustard	modal chernozem	**↓** Decreased Cd and Zn mobility	**↓** Decreased Cd and Zn uptake, **↑** Increased Pb accumulation, ↑ Increase biomass	Heavy metal adsorption by biochar, increased plant tolerance by increasing glutamic acid and glutamine	[137]
Rice straw biochar 300 °C	Pb	No plants	utisol and oxisol	**↓** Decreased availability of Pb	No plants	Enhanced negative charge of soil and contributed to the non-electrostatic adsorption of Pb (II); formation of surface complex between oxygen-containing functional groups on biochar with Pb (II)	[138]
Poplar wood biochar 525 °C	Cu	Sunflower	Cu-contaminated soil	Significantly reduced leachable Cu	**↑** Increased plant biomass and nutrient (K) concentration, plant height, and root length. **↓** Reduced shoot Cu concentration	Decreased Cu bioavailability, improved soil nutrient and water provision Nutrients (Ca) compete with Cu for plant uptake	[139]
Mixed wood biochar	Cu and Pb	Ryegrass	heavily Cu- and Pb-contaminated soil	**↓** Reduced pore water Cu concentration, ↓ reduced shoot	**↓** Reduced Cu and Pb uptake by ryegrass shoots, **↑** Increased biomass	Humified complexes and the formation of minerals such as pyromorphite (with P-rich biochar) reduced Cu in pore water, high pH reduced Pb in pore water	[140]
Willow biochar, wheat straw biochar	Cd, Pb, Zn, Cr, Cu, and Ni	*Lepidium sativum*	heavy-metal-contaminated soil	Biochar ↓ reduced the toxicity of soil leachates	Eliminated root growth inhibition	Adsorption of heavy metals	[80]
Pinewood biochar	Cd	Lotus	Cu-polluted water–soil	Not measured	**↑** Increased total and rhizome biomass, **↓** Decreased Cd content in rhizomes, petioles, and leaves, increased Cd transfer efficient from underground to aboveground tissues, **↓** Reduced Cd content in edible part	Biochar formed insoluble chelates or caused precipitation of Cd and alleviate Cd stress, reducing SOD activity of lotus plant	[141]
Cotton wood biochar 600 °C	Ni	Tomato	hydroponic system	Not measured	Alleviated fruit yield reduction by 26.6%, minimized the reduction in nutrients concentrations in roots, shoots, and fruits	Precipitation of Ni on biochar in crystal form, ion exchange, and complexation with surface functional groups of biochar; alleviated heavy metal stresses on tomato (distortion of nucleolus, thickening formation in cell wall structure, reduction in chlorophyll content, vacuolization)	[142]
Chicken manure biochar and green waste biochar 550 °C	Cd, Cu, and Pb	Indian mustard	metal-spiked soils and naturally metal-contaminated soils	**↓** Reduced NH4NO3 extractable Cd, Cu, and Pb in soils, **↓** Decreased Cd and Cd and Pb but increased Cu concentration in pore water	**↑** Increased shoot and root biomass by 353% and 572% (chicken waste biochar), **↓** Reduced Cd, Cu, and Pb accumulation by Indian mustard, and **↑** Increased nutrient availability of P and K	Metal immobilization by biochar with both specific (coulombic interaction) and non-specific (coordination bonds) adsorption; changed the partitioning of Cd, Cu, and Pb from easily exchangeable phase to organic-bound fraction	[143]
Sugarcane straw biochar 700 °C	Cd, Cu, Pb, and Zn	Jack bean and *Mucuma aterrima*	contaminated Zn mine soil	**↓** Reduced Zn in pore water	**↓** Reduced plant uptake of Cd, Pb, and Zn	Reduced heavy metal toxicity and increased macronutrient (P, K, Ca, and Mg) concentrations in soil	[144]
Cattle manure biochar 400 °C	A1	Wheat	solution	**↓** Reduced Al3+ concentration in solution	**↓** Reduced Al uptake by wheat, enhanced root and shoot elongation, avoided root tip plasma membrane damage	Biochar elevated solution pH, facilitated the transfer of free Al3+ ions to Al(OH)2+ and Al(OH)2+ monomers, which were adsorbed by biochar through surface complexation rather than electrostatic attraction (between Al3+ and biochar negative surface charge)	[145]
Sewage sludge, soybean and rice straw, peanut shell biochar 500 °C	Cd, Cu, Pb, and Zn	Turnip	As, heavy metal, and PAHs combined	**↓** Decreased bioaccessibility of Cd, Cu, Pb, and Zn concentration	**↑** Increased root yield by 2% application rate biochar, **↓** Decreased root yield by 5% application rate biochar, **↓** Reduced plant heavy metal accumulation	Precipitation of heavy metal ions (at high pH) with different anions (OH-, SO 2-, HPO-, CO 2-) in soil and O-functional groups in biochar, e.g., forming metal–P precipitates.	[146]
**Hazardous metalloid**							
Mixed wood biochar 400 °C	As	Miscanthus	As-contaminated soil	Little effects on As mobility	Little effects on plant yield and As uptake	Increased P availability, P competed with As for the uptake site	[147]
Sewage sludge, soybean and rice straw, peanut shell biochar 500 °C	As	Turnip (*Brassica rapa* L.)	As, heavy metal, and PAHs combined contaminated soil	**↓** Decreased bioaccessibility of As concentration (more with 5% than 2% biochar)	**↑** Increased root yield by 2% application rate biochar, **↓** Decreased root yield by 5% application rate biochar, **↓** Reduced plant As accumulation	P competed with As for plant uptake; Si chelated As; S reduced As availability; 5% biochar application rate increased NH4 +-N availability, causing stress response of plant	[146]
**Organic contaminants**							
Willow biochar, wheat straw biochar	PAHs	*Lepidium* *sativum*	PAHs-contaminated soil	Biochar **↓** Reduced the toxicity of soil leachates	Eliminated root growth inhibition	Adsorption of PAHs	[80]
Pine chip biochar 500 °C	DDT, DDE, and deldrin residue	Orchard grass	old orchard soil with continuous DDT application	Ineffective in lower bioavailability factor of organochlorine pesticide residues	**↑** Increased shoot biomass	Possibly due to low surface areas of biochar in use	[148]
Wood charcoal (produced in an earthen pit) and eucalyptus wood biochar 800 °C	S-metolachlor and sulfentrazone	Green Foxtail (*Setaria viridis*)	Hamra Red Mediterranea n subsoil	**↓** Reduced the bioavailability of herbicides to the weed	Biochar alleviated the inhabitation of weed biomass with the use of herbicides, biochar with high SSA **↓** Reduced herbicide efficacy	High SSA caused high adsorption of herbicides	[105]
Sewage sludge, soybean and rice straw, peanut shell biochar 500 °C	PAHs	Turnip (*Brassica rapa* L.)	As, heavy metal, and PAHs combined contaminated soil	**↓** Decreased the accessible concentrations of ∑16PAHs	**↓** Reduced PAH concentrations in turnip, higher reduction for high-molecular-weight PAHs	High surface area, low polarity, and high C content contributed to PAHs adsorption	[146]
Wheat straw biochar 500 °C	Hexachlorobe nzene (HCB)	Ryegrass	ferri-udic Argosols	**↓** Reduced HCB bioavailability to microbes and plants, and **↓** Reduced microbial degradation	**↓** Reduced HCB concentration in shoots and roots	Adsorption of HCB by biochar; ryegrass root exudates (oxalic acid) suppressed HCB sorption to biochar and stimulated HCB microbial rhizodegradation	[149]
Wheat straw biochar 300 °C	Ionizable herbicide (4-chloro-2-m ethylphenoxy) acetic acid (MCPA)	Sunflower	soil with 60% silt content	**↓** Increased MCPA sorption and **↓** Decreased desorption, leachability, and dissipation (microbial degradation)	No significant effects in terms of the phytotoxic effects of MCPA on sunflower, biochar **↑** Increased aboveground biomass but **↓** Reduced chlorophyll a,b content	Partition of MCPA to biochar; Mg deficiency may be ascribed to the reduced chlorophyll content	[150]
Wheat chaff biochar 450 °C and wood biochar (eucalyptus) 450 °C and 520 °C	Carbamazepin e (CBZ) and propranolol (PRL)	Ryegrass	loamy sand soil	**↓** Reduced active pharmaceutical ingredients (APIs) concentration in pore water	**↓** Reduced plant tissue uptake of APIs	Both partitioning of APIs to biochar amended soil (natural solids in soil) and sorption of APIs to biochar	[151]
Cotton straw chips 450 °C and 850 °C	Insecticides (chlorpyrifos and fipronil)	Chinese chives	clay loamy soil (pH = 4.01)	↑ Increased the half-life of insecticides **↓** Lowered the availability of insecticides to soil microbes	**↓** Reduced plant uptake and microbial degradation **↓** Reduced pesticide residues in both aboveground and underground plant parts **↑** Increased plant biomass	High surface area and microporosity; biochar sequestration and microbial degradation	[152]
Eucalyptus spp. wood chips 450 °C and 850 °C	Insecticides (chlorpyrifos and carbofuran)	Spring onion	sand loamy soil	**↓** Decreased the extractable insecticide residues in soil	**↑** Higher biomass under biochar amendment, **↓** Lower insecticide residues in above- and belowground plant parts	Biochar sequestration and microbial degradation	[153]
